# Active Versus Passive Cancer Registry Methods Make the Difference: Case Report From Colombia

**DOI:** 10.1200/JGO.17.00093

**Published:** 2017-08-28

**Authors:** Esther de Vries, Constanza Pardo, Carolina Wiesner

**Affiliations:** **Esther de Vries**, Pontificia Universidad Javeriana; **Constanza Pardo** and **Carolina Wiesner**, National Cancer Institute, Bogotá, Colombia

## TO THE EDITOR:

As an institute and as professionals working on a daily basis to improve prevention, detection, and treatment of cancer in Colombia, we were interested to read the recent article in *Journal of Global Oncology* by Valencia et al^1^ on how methodology influences cancer incidence and prevalence data, in particular in our country.

It makes complete sense that prevalence data vary widely between the GLOBOCAN methods and the Cuenta de Alto Costo (CAC). The first is on the basis of models that calculate prevalence as a result of regional observed incidence data and cancer survival rates from other countries, and the CAC relies on passive reporting by patients’ private insurance companies (EPS). If all patients are known by the EPS and their reports achieve 100% coverage, then the CAC prevalence data should be correct.

Differences in incidence rates between the CAC and the population-based cancer registries (PBCR) are not as easy to understand. If the reporting of new cases by the EPS to the CAC is (almost) complete, then incidence rates should be close to those reported by the existing PBCR in the countries; lower rates most likely indicate underreporting. Rather than comparing to the GLOBOCAN (estimated) data, in Table 1 we list a sample of important cancer types and their observed age-standardized incidence rate according to the CAC data versus the PBCR data. We assumed that both the CAC data and the PBCR data were standardized using the Segi world population^5^; unfortunately, the standard population used was not specified in any of the publications.^1,6^

**Table 1:**
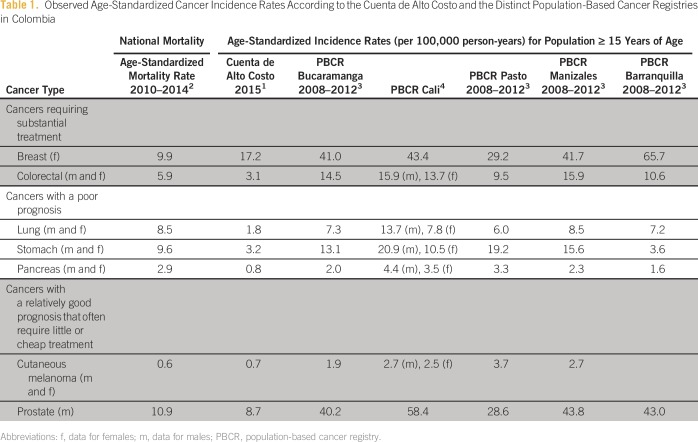
Observed Age-Standardized Cancer Incidence Rates According to the Cuenta de Alto Costo and the Distinct Population-Based Cancer Registries in Colombia

The observed incidence rates of the CAC are consistently lower than those of the PBCR, even when compared with data from the PBCR of Barranquilla, which is probably not 100% complete and therefore prudently does not yet publish its data. For most localities listed in Table 1, the CAC incidence rates are even lower than the mortality rates, even in cancers with good prognoses. The most probable reason for the discrepancies is, indeed, in the methodology used, ie, passive case reporting by the EPS (CAC) versus active case finding by PBCR personnel.^7^ The main arguments for the quality and completeness of the CAC data provided by Valencia et al are as follows: (1) the revision of patient and tumor data, and (2) the fact that reporting of cancer cases by the EPS to the CAC is mandatory.^1^ The first argument does not by any means influence completeness of population coverage, and the second has repeatedly been shown to be insufficient to reach good coverage.^7^ The fact that the discrepancies between the CAC and the PBCR are largest for either cancers with a poor prognosis or those that require little or cheap treatment shows the danger of relying on cost arguments in assuming that reporting is complete. In a country with long wait times and administrative rules, many patients (even those with relatively low incomes) would prefer to pay out of pocket for excision of a localized melanoma or for follow-up visits in a wait-and-see management approach to prostate cancer, rather than rely on the system. The management of cancers diagnosed close to the time of death is usually inexpensive, and therefore the incentives for reporting by the EPS are minimal. Moreover, many EPS do not have good insight into the number of patients with cancer with whom they are affiliated, invalidating all assumptions regarding the reporting mechanisms. This also explains why the breast and colorectal cancer estimates are so disparate—the EPS probably do not report all of their cases.

Upon inspecting both incidence data of the CAC versus the PBCR and estimated data by the International Agency for Research of Cancer, one thing is clear: The CAC incidence data are > 50% lower than those observed regionally and calculated for the country. The age-standardized incidence of 45.6 is 30 per 100.000 lower than the national cancer mortality rate for 2008 to 2012 (75.5 per 100.000^3^). If the data of the CAC really provide “a more precise real-world estimation of new cases each year,”^1^ then Colombia has something special on a global level—among the lowest incidence rates worldwide. For the moment, the National Cancer Institute will continue to rely on the PBCR data.
